# Single-Molecule Real-Time Transcript Sequencing of Turnips Unveiling the Complexity of the Turnip Transcriptome

**DOI:** 10.1534/g3.120.401434

**Published:** 2020-08-07

**Authors:** Hongmei Zhuang, Qiang Wang, Hongwei Han, Huifang Liu, Hao Wang

**Affiliations:** Institute of Horticultural Crops, Xinjiang Academy of Agricultural Sciences, Urumqi 830091, Xinjiang, China

**Keywords:** turnips (*Brassica rapa var. Rapa*), single-molecule real-time sequencing, full-length transcriptome

## Abstract

To generate the full-length transcriptome of Xinjiang green and purple turnips, *Brassica rapa var. Rapa*, using single-molecule real-time (SMRT) sequencing. The samples of two varieties of *Brassica rapa var. Rapa* at five developmental stages were collected and combined to perform SMRT sequencing. Meanwhile, next generation sequencing was performed to correct SMRT sequencing data. A series of analyses were performed to investigate the transcript structure. Finally, the obtained transcripts were mapped to the genome of *Brassica rapa ssp. pekinesis* Chiifu to identify potential novel transcripts. For green turnip (F01), a total of 19.54 Gb clean data were obtained from 8 cells. The number of reads of insert (ROI) and full-length non-chimeric (FLNC) reads were 510,137 and 267,666. In addition, 82,640 consensus isoforms were obtained in the isoform sequences clustering, of which 69,480 were high-quality, and 13,160 low-quality sequences were corrected using Illumina RNA seq data. For purple turnip (F02), there were 20.41 Gb clean data, 552,829 ROIs, and 274,915 FLNC sequences. A total of 93,775 consensus isoforms were obtained, of which 78,798 were high-quality, and the 14,977 low-quality sequences were corrected. Following the removal of redundant sequences, there were 46,516 and 49,429 non-redundant transcripts for F01 and F02, respectively; 7,774 and 9,385 alternative splicing events were predicted for F01 and F02; 63,890 simple sequence repeats, 59,460 complete coding sequences, and 535 long-non coding RNAs were predicted. Moreover, 5,194 and 5,369 novel transcripts were identified by mapping to *Brassica rapa ssp. pekinesis* Chiifu. The obtained transcriptome data may improve turnip genome annotation and facilitate further study of the *Brassica rapa var. Rapa* genome and transcriptome.

Brassica crop renowned for their wide genetic and diverse phenotypes. *B. rapa* (AA, 2n = 20), *B. oleracea* (CC, 2n = 18), and *B. napus* (AACC, 2n = 38) are the three most well known Brassica crops (Wang *et al.* 2011; Lin *et al.* 2014). Several subspecies in *B. rapa* have been cultivated for particular phenotypic characteristics, including turnips, Chinese cabbage, broccoli, cauliflower, and the oilseed field mustard (Qi *et al.* 2017). Turnips (*Brassica rapa* ssp. rapa) represent an important morphological type of *B. rapa* species, cultivated in Europe since 2,500-2,000 B.C. and spread to other parts of the world afterward (Gray 1883; Zhang *et al.* 2014; Qi *et al.* 2017). Turnips are cultivated as fodder crop or vegetables, and its leaves and tubers are consumed depending on the region. The shoots and leaves are consumed in southern European countries, while fleshy root in northern and eastern Europe and China (Zhang *et al.* 2014).

Turnips (*Brassica rapa* ssp. rapa), known to residents of Xinjiang as Qiamagu, are also known as rutabaga, round root, and dish plant, among other names. Xinjiang turnips are a cruciferous *brassica* biennial herbaceous plant belonging to the same species of inland turnips while differing greatly in shape and flavor (Ma *et al.* 2016). Turnips are a characteristic indigenous vegetable commonly eaten by people of all ethnic groups in Xinjiang. Residents eat the fat fleshy roots, which have a high nutrient content and are rich in protein and mineral elements (Shi *et al.* 2011). In many areas of southern Xinjiang, turnip is regarded as an indispensable food and is eaten every day. Turnips are considered by local ethnic minorities to be as important as ginseng is to the Han people, who regard ginseng as the “holy fruit of longevity” (Kortesniemi *et al.* 2015). In addition to being nutrient-rich, turnips have been shown to have medicinal value, and all the properties of Xinjiang turnips, such as its taste, functions, usage, and dosage, are recorded in detail in the Chinese Materia Medica Volume 4 (Fernandes *et al.* 2007; Li 2018). For the above reasons, the turnip is widely planted in Xinjiang, and the production area has increased from year to year. Structural and functional genomics are the basis for understanding plant biology (Saito and Matsuda 2010). Wang *et al.* previously reported the annotation and analysis of the draft genome sequence of *Brassica rapa ssp. pekinesis* Chiifu (https://www.ncbi.nlm.nih.gov/genome/?term=Brassica+rapa) in 2011 (Wang *et al.* 2011). Turnip is one of the subspecies of *Brassica rapa*. However, to date, the genome and transcriptome of *Brassica rapa var. Rapa* have not been investigated.

The transcriptome can reflect changes in gene expression during various physiological and biochemical processes (Yang *et al.* 2014). Hence, different transcriptome sequencing techniques have been developed and applied with various merits and demerits, of which short-read sequencing is considered as a potent method for profiling the transcriptome (Yang *et al.* 2018). Nevertheless, these techniques are mostly unsuitable for the analysis of specific biological processes due to the limitations of short read lengths, including assembly, detection of gene isoforms, and complex genomic regions (Rhoads and Au 2015). These limitations can be overcome by using single-molecule real-time (SMRT) sequencing, which was developed by Pacific BioSciences (PacBio) (Roberts *et al.* 2013). This approach uses real-time sequencing without the requirement of pausing between read steps, and is categorized as third-generation sequencing (Schadt *et al.* 2010).

SMRT can process read lengths of more than 20 kb for full-length transcripts or longer length fragments (Rhoads and Au 2015). Full-length transcripts can greatly elevate the veracity in annotating the genome and in characterizing the transcriptome, and can be used for the analysis of exon-intron structure and alternative splicing, which is helpful to completely understand the transcriptional behavior of genomic loci (Dong *et al.* 2015). Alternative splicing (AS) is one of the common ways to diversify the functional characteristics of the transcriptome and proteome in eukaryotic organisms (Reddy *et al.* 2013; Kalsotra and Cooper 2011). AS has been implicated in the regulation of plant development because it occurs in approximately 40–60% of intron-containing transcripts in different tissues and developmental stages in *Arabidopsis thaliana* (Marquez *et al.* 2012), *Zea mays* (Thatcher *et al.* 2014), and *Oryza sativa* (Dong *et al.* 2018).

Most previous research on the Xinjiang turnip has focused on its medical physiology, such as its content of linoleic acid, flavonoids, saponins, vitamins (B, C, and PP), calcium, iron, and other effective elements, nutrients, and amino acids. However, there has been little further investigation into *Brassica rapa var. Rapa*. Therefore, SMRT sequencing was used in this study to generate the full-length transcriptome of two *Brassica rapa ssp. Rapa* turnips varieties (Xinjiang purple turnip and green turnip, named based on their root skin colors), followed by analysis of simple sequence repeat (SSR) and AS, and prediction of coding sequence and long non-coding RNA (lncRNA). This study was expected to improve the annotation of the turnip genome (*Brassica rapa var. Rapa*) and to provide a valuable resource for basic research and the molecular breeding of turnips.

## Materials and Methods

### Workflow presentation

The analysis process of this study is shown in Figure S1. Briefly, the third generation transcriptome sequencing data were used to assemble a complete transcript for structural optimization, while the second-generation transcriptome data were used for correction and quantitative analysis of the third generation transcriptome data (Figure S1).

### Plant materials and RNA sample preparation

**Figure 1:**
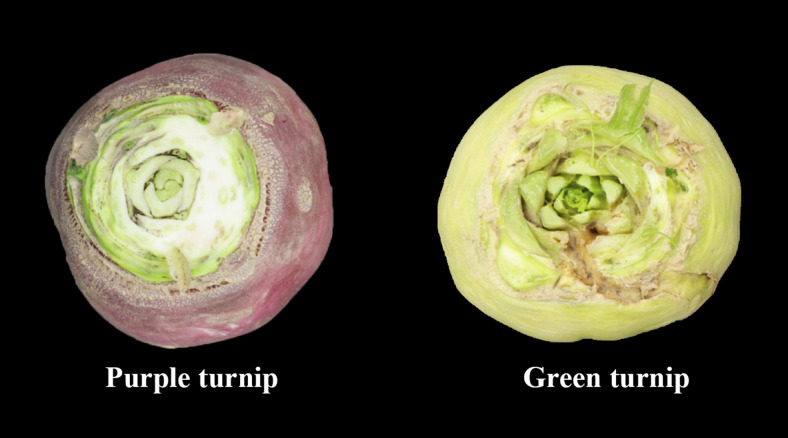
The phenotypes of the fleshy root of green and purple turnips.

Green and purple *Brassica rapa var. Rapa* turnips were planted and grown under the same conditions in the experimental greenhouse at the Comprehensive Laboratory of the Xinjiang Academy of Agricultural Sciences (Xinjiang, China). Skin samples (phloem and periderm) were collected from the fleshy root of turnips using a knife blade at five stages during the vegetative period of green (F01) and purple turnips (F02), including the seedling stage, the succulent root enlargement stage, and the early, middle, and mature periods of succulent root expansion. The phenotypes of the fleshy root of green and purple turnips are shown in Figure 1. Three biological replicates were used, and a total of 15 samples were collected from each strain. Total RNA isolation was conducted using the RNeasy Plus Mini Kit (Qiagen, Valencia, CA, USA), and their purity and concentration were measured using an OD260/280 reading on a NanoDrop ND-1000 spectrophotometer (NanoDrop Technologies, Rockland, DE, USA). The integrity assessment was conducted utilizing the RNA Nano 6000 Assay Kit on an Agilent Bioanalyzer 2100 system (Agilent Technologies, CA, USA). The total RNA samples of five developmental stages from each strain were mixed for the following analysis.

### PacBio Iso-Seq library construction and sequencing

The isoform-sequencing (Iso-seq) library was constructed based on the protocol described by Pacific Biosciences (P/N00-377-100-05 and P/N100-377-100-04). The full-length mRNA cDNA was synthesized using the SMARTerTM PCR cDNA synthesis kit. Then, the BluePippin was used to screen full-length cDNA fragments and build cDNA libraries of different sizes. Next, the full-length cDNA was amplified and extended by PCR, followed by terminal repair. The cDNA was connected to the SMRT dumbbell-type connector, and exonuclease digestion was performed. BluePippin was used for secondary screening to obtain the sequencing library. The accurate quantification of libraries was performed using Qubit 2.0, followed by the detection of library size using Agilent 2100. The libraries were then sequenced on PacBio RSII according to the target data volume. The PacBio RSII database binned lengths of 0-1 kb, 1-2 kb, 2-3 kb, 3-6 kb, and >6 kb. 2 turnips and 5 vegetative growth stages were analyzed, and 18 cells were used in total.

### Illumina cDNA library construction and sequencing

Purification of mRNA from 3 μg total RNA of each sample was completed utilizing poly-T oligo-attached magnetic beads, followed by fragmentation using fragmentation buffer. Then, the purified mRNA was used to synthesize cDNA using random hexamers (first strand), Nase H, and DNA polymerase I (second strand). After purification, end repairing, adenylation, and adapter ligation, the cDNA fragments were enriched by PCR amplification, and the cDNA libraries were constructed and sequenced on an Illumina HiSeq 2500 platform (Illumina Inc., San Diego, CA, USA).

### Pacific biosciences long-read processing

Raw reads were processed into error-corrected reads of insert (ROIs) according to the conditions full passes ≥ 0 and sequence accuracy > 0.75. By detecting whether each ROI sequence contains a 5′ primer, 3′ primer, and poly-A tail, the sequences can be divided into full-length sequences (including all three elements) and non-full-length sequences. Based on the poly-A tail signal and the 5′ and 3′ cDNA primers in ROIs, the full-length non-chimeric (FLNC) transcripts were identified. The ICE algorithm in SMRT Analysis (v2.3.0) software was used to obtain a consistent sequence (consensus) isoform, and the full-length consensus sequences were polished using Quiver. Full-length transcripts with post-correction accuracy above 99% (high-quality isoforms) were generated for further analysis. The redundant sequences were removed from the high-quality and corrected low-quality transcript sequences of each sample using CD-HIT software (Li and Godzik 2006).

### AS detection

The three-generation transcripts were mapped onto *Brassica rapa* genome (http://brassicadb.org/brad/datasets/pub/BrassicaceaeGenome/Brassica_rapa/V3.0/) using gmap with parameters setting as: similarity = 0.9 and Coverage = 0.85. Then AS events were analyzed using Astalavista.

### SSR detection

MISA (MIcroSAtellite identification tool, http://pgrc.ipk-gatersleben.de/misa/) is a software used to identify SSRs, and it can identify 7 types of SSR by analyzing transcript sequences. SSR analysis was performed on transcripts more than 500 bp long using MISA.

SSR detection was analyzed using MISA based on the following criteria:

Definition (unit_size,min_repeats): 1-10 2-6 3-5 4-5 5-5 6-5

Interruptions (max_difference_between_2_SSRs): 100

For the definition of SSR, mono-nucleotide repeats 10 times or more; di-nucleotide repeats 6 times or more; tri-nucleotide, tetra-nucleotide, penta-nucleotide and hexa-nucleotide repeats 5 times or more. The distance between two SSRs is less than 100 bp and is defined as SSR compliant.

### Prediction of long non-coding RNAs (lncRNAs)

The lncRNAs were predicted by analyzing the coding potential of transcripts using four widely used computational approaches, including the coding potential calculator (CPC) (Kong *et al.* 2007), coding-non-coding index (CNCI) (Sun *et al.* 2013), coding potential assessment tool (CPAT) (Wang *et al.* 2013), and Pfam protein structure domain analysis. Furthermore, the target transcripts of the obtained lncRNAs were predicted using the LncTar tool (Haas *et al.* 2013).

### Prediction of coding sequence (CDS)

TransDecoder is a software used to predict CDSs, and can identify potential CDSs from transcript sequences based on the alignment of open reading frame length and the log-likelihood score of amino acid sequences to protein domain sequences in the Pfam database. The prediction of CDSs and corresponding amino acid sequences was performed using TransDecoder (version 3.0.0) (Xanthopoulou *et al.* 2016).

### Functional annotation of transcripts

Functional annotation was performed for the non-redundant transcript sequences by mapping to NR, Swissprot, Gene Ontology (GO), Clusters of Orthologous Groups of proteins (COG), euKaryotic Ortholog Groups (KOG), Protein Family (Pfam), and KEGG databases using BLAST (version 2.2.26).

### Transcript mapping to field mustard (Brassica rapa)

The published representative genome for field mustard (*Brassica rapa*) (assembly Brapa_1.0) was downloaded from NCBI as the reference transcriptome to identify potential novel transcripts. Next, the obtained transcripts were mapped to the reference transcriptome using BLASR, a long sequence alignment software commonly used in third-generation sequencing. The parameter was set as -bestn 1, and other parameters were set as default. The mapping rate (the percentage of mapped bases to the total number of bases in the sequence) and matching rate (the percentage of exactly matched bases to the total number of mapped bases) were calculated, and the known transcripts were determined to have mapping and matching rates over 70%, otherwise the transcripts were defined as novel transcripts.

### Data Availability Statement

The raw data were available at NCBI Sequence Read Archive (SRA) repository with Accession Number SRP218278. Supplemental material available at figshare: https://doi.org/10.25387/g3.11790984.

## Results

### SMRT sequencing statistics

PacBio RSII was used in SMRT sequencing data output mode. For Xinjiang green turnip (F01), after data preprocessing from 1,202,336 polymerase reads (Table 1), a total of 19.54 Gb clean data were obtained in 8 cells. A total of 510,137 ROIs were screened based on the data filtering criteria described in the methods section (Table 2). Additionally, 267,666 FLNC sequences were screened. The percentage of full-length transcripts was more than 46.35%, and the percentage of artificial concatemers sequences was 0.42%, suggesting a moderate SMRTbell concentration (Table 3).

**Table 1:** Polymerase reads sequence statistics

Sample Name	Sample ID	cDNA size	SMRT Cells	Polymerase Reads	Post-Filter Polymerase Reads	Post-Filter Total Number of Subread Bases	Post-Filter Number of Subread	Post-Filter Subreads N50	Post-Filter Mean Subread length
F01	F01	1-2K	3	450876	260542	6938403141	4596750	1476	1509
F01	F01	2-3K	3	450876	283475	6553426599	2584540	2514	2535
F01	F01	3-6K	2	300584	239349	6044701111	2025161	3235	2984
F02	F02	1-2K	3	450876	308304	8032628518	5107420	1538	1572
F02	F02	2-3K	3	450876	303904	7074361017	2569287	2709	2753
F02	F02	3-6K	2	300584	230942	5303537918	1469284	3663	3609

cDNA size: insert fragment size of cDNA libraries; SMRT cells: the number of cells used for library construction; Polymerase reads: the number of polymerase read sequences after sequencing; Post-filter polymerase reads: the number of polymerase read sequences after filtration; Post-filter total number of subread bases: the number of subread bases after filtration; Post-filter number of subreads: the number of subreads after filtration; Post-filter subreads N50: subread N50 length after filtration; Post-filter mean subread length: average length of subread after filtration.

**Table 2:** Reads of insert (ROI) statistics

Samples	cDNA size	Reads of Insert	Read Bases of Insert	Mean Read Length of Insert	Mean Read Quality of Insert	Mean Number of Passes
F01	1-2K	197,712	400,090,514	2,023	0.94	16
F01	2-3K	185,044	493,389,501	2,666	0.93	10
F01	3-6K	127,381	403,940,195	3,171	0.92	9
F01	All	510,137	1,297,420,210	2,542	0.93	12
F02	1-2K	230,897	436,463,585	1,890	0.94	15
F02	2-3K	180,575	520,488,291	2,882	0.92	9
F02	3-6K	141,357	488,240,163	3,453	0.91	8
F02	All	552,829	1,445,192,039	2,613	0.92	11

cDNA size: insert fragment size of cDNA libraries; ROIs: the number of ROI sequences; Total ROI bases: the total number of ROI bases; Mean ROI length: average length of ROI; Mean ROI quality: Quality value of ROI sequence; Mean number of passes: the mean sequencing depth of sequences in zero-mode wave.

**Table 3:** Full-length sequence statistics

Samples	cDNA Size	Reads of Insert	Number of five prime reads	Number of three prime reads	Number of poly-A reads	Number of filtered short reads	Number of non-full-length reads	Number of full-length reads	Number of full-length non-chimeric reads	Average full-length non-chimeric read length	Full-Length Percentage (FL%)	Artificial Concatemers (%)
F01	1-2K	197,712	122,794	133,392	129,503	24,318	67,847	105,547	104,764	1,231	53.38%	0.74%
F01	2-3K	185,044	121,426	127,171	125,657	10,998	69,849	104,197	103,967	2,290	56.31%	0.22%
F01	3-6K	127,381	73,951	77,972	76,208	8,729	59,607	59,045	58,935	2,906	46.35%	0.19%
F01	All	510,137	318,171	338,535	331,368	44,045	197,303	268,789	267,666	2,011	52.69%	0.42%
F02	1-2K	230,897	130,863	147,167	142,222	36,777	85,056	109,064	108,466	1,242	47.23%	0.55%
F02	2-3K	180,575	104,783	111,538	110,080	14,477	79,286	86,812	86,660	2,300	48.08%	0.18%
F02	3-6K	141,357	91,815	95,442	94,654	4,468	56,962	79,927	79,789	3,299	56.54%	0.17%
F02	All	552,829	327,461	354,147	346,956	55,722	221,304	275,803	274,915	2,172	49.89%	0.32%

cDNA size: insert fragment size of cDNA libraries; Reads of insert: the number of ROI sequences; Number of 5′ reads: the number of ROI sequences containing 5′ ends; Number of 3′ reads: the number of ROI sequences containing 3′ ends; Number of poly-A reads: the number of ROI sequences containing poly-A sequence; Number of filtered short reads: the number of filtered ROIs of <300 bp; Number of non-full-length reads: the number of non-full-length ROIs; Number of FLNC reads: the number of full-length non-chimeric ROIs;Average FLNC read length: average length of full-length non-chimeric sequences; Full-length percentage (FL%): the percentage of ROIs that were full-length sequences;Artificial concatemers (%): the percentage of ROIs that were full-length chimeric sequences.

Similarly, for Xinjiang purple turnip (F02), there were 1,202,336 polymerase reads (Table 1), and a total of 20.41 Gb clean data were obtained in 8 cells. In addition, 552,829 ROIs (Table 2) and FLNC sequences were obtained. The percentage of full-length transcripts was more than 47.23%, and the percentage of artificial concatemers sequences was 0.32%, suggesting a moderate SMRTbell concentration (Table 3).

### Clustering analysis and correction of isoform sequences

In this study, using the SMRT analysis software RS_IsoSeq module, clustering analysis was carried out on the total length of the sequence, and the lengths of the transcriptome data for two different species of turnips were compared. From the Xinjiang green turnip (F01), 82,640 consensus isoforms were obtained, of which high-quality isoforms (69,480 high-quality isoforms) accounted for 84.08%. In addition, 13,160 low-quality isoforms were obtained, which were corrected using Illumina RNA-seq to improve the sequence accuracy (Table 4).

**Table 4:** Results of Iterative Clustering for Error Correction (ICE) clustering analysis

Samples	Size	Number of consensus isoforms	Average consensus isoforms read length	Number of polished high-quality isoforms	Number of polished low-quality isoforms	Percent of polished high-quality isoforms (%)
F01	0to1kb	9,164	879	8,568	596	93.50%
F01	1to2kb	29,434	1,392	25,900	3,534	87.99%
F01	2to3kb	34,914	2,424	29,270	5,644	83.83%
F01	3to6kb	8,117	3,548	5,724	2,393	70.52%
F01	above6kb	1,011	9,556	18	993	1.78%
F01	All	82,640	2,082	69,480	13,160	84.08%
F02	0to1kb	10,600	894	10,003	597	94.37%
F02	1to2kb	33,144	1,393	29,904	3,240	90.22%
F02	2to3kb	28,963	2,397	24,579	4,302	84.86%
F02	3to6kb	20,566	3,454	14,245	5,594	69.26%
F02	above6kb	1,329	9,048	67	1,244	5.04%
F02	All	94,602	2,200	78,798	14,977	83.29%

For Xinjiang purple turnip (F02), 93,775 consensus sequences were obtained, of which 78,798 were high-quality isoforms, accounting for 83.29%. A total of 14,977 low-quality isoforms were obtained, which were corrected using Illumina RNA-seq (Table 4). The redundant sequences from the high-quality and the corrected low-quality transcript sequences were removed using CD-HIT. A total of 46,516 non-redundant sequences were obtained for F01, and 49,429 non-redundant sequences were obtained for F02. Then, sequences that were common to both samples were combined, and a total of 83,820 non-redundant transcript sequences were obtained.

### AS detection

**Figure 2:**
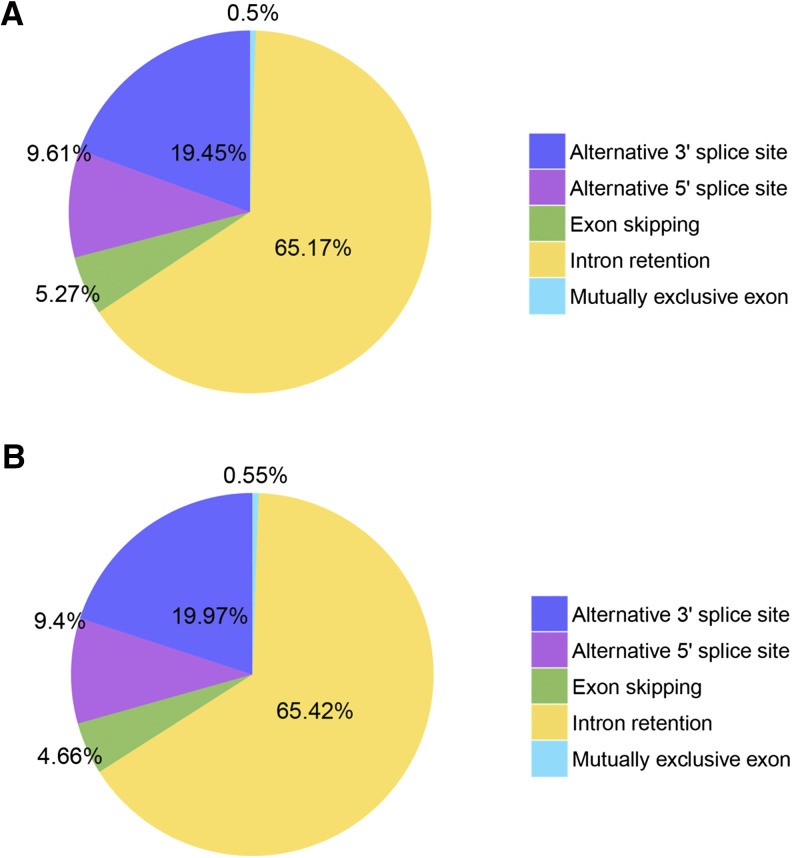
Alternative splicing events statistics The pie chart showing the results of alternative splicing events statistics in F01 (A) and F02 (B).

Using *Brassica rapa* genome (http://brassicadb.org/brad/datasets/pub/BrassicaceaeGenome/Brassica_rapa/V3.0/) as a reference genome, AS events were identified. A total of 7,774 AS events were identified in Xinjiang green turnips (F01) (Table S1), including 39 mutually exclusive exon, 5,066 intron retention, 410 exon skipping, 747 alternative 5′ splice site, and 1,512 alternative 3′ splice site (Figure 2A). For Xinjiang purple turnips (F02), a total of 9,385 AS events were identified (Table S2), including 52 mutually exclusive exon, 6,140 intron retention, 437 exon skipping, 882 alternative 5′ splice site and 1,874 alternative 3′ splice site (Figure 2B).

### SSR detection

**Figure 3:**
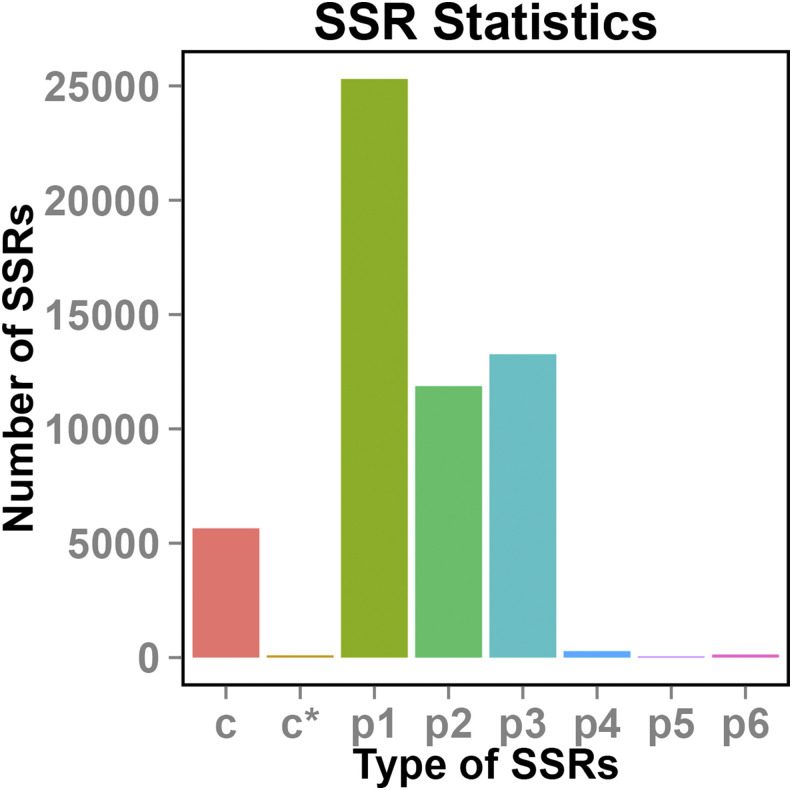
SSRs statistics c, there is no base sharing between two SSRs; c*, there is base sharing between two SSRs; p1, perfect mono-nucleotide repeat; p2, perfect di-nucleotide repeat; p3, perfect tri-nucleotide repeat; p4, perfect tetra-nucleotide repeat; p5, perfect penta-nucleotide repeat; p6, perfect hexa-nucleotide repeat.

In all, 83,767 transcript sequences (total 175,104,191 bp) were used to evaluate the SSR, and a total of 63,890 SSRs were identified. In addition, 40,271 SSR-containing sequences, 14,540 sequences containing more than one SSR, and 7238 SSRs that exist as complexes were detected. Moreover, it was found that mononucleotide repeats were the most common type of SSR, followed by dinucleotide repeats and trinucleotide repeats (Figure 3).

### Prediction of CDS

**Figure 4:**
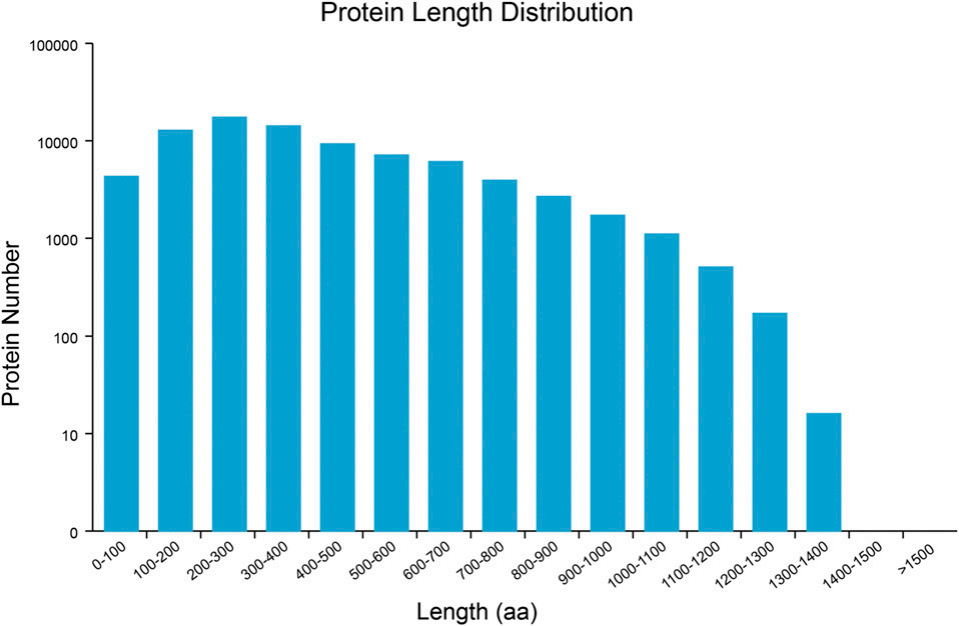
Distribution of the coding sequence lengths of complete ORFs The x-axis represents the coding sequence length, and the y-axis represents the number of predicted ORFs.

According to the method described above, a total of 81,371 open reading frames were identified, including 59,460 complete open reading frames. The protein sequence length of complete open reading frames is shown in Figure 4.

### Functional annotation of transcripts

**Figure 5:**
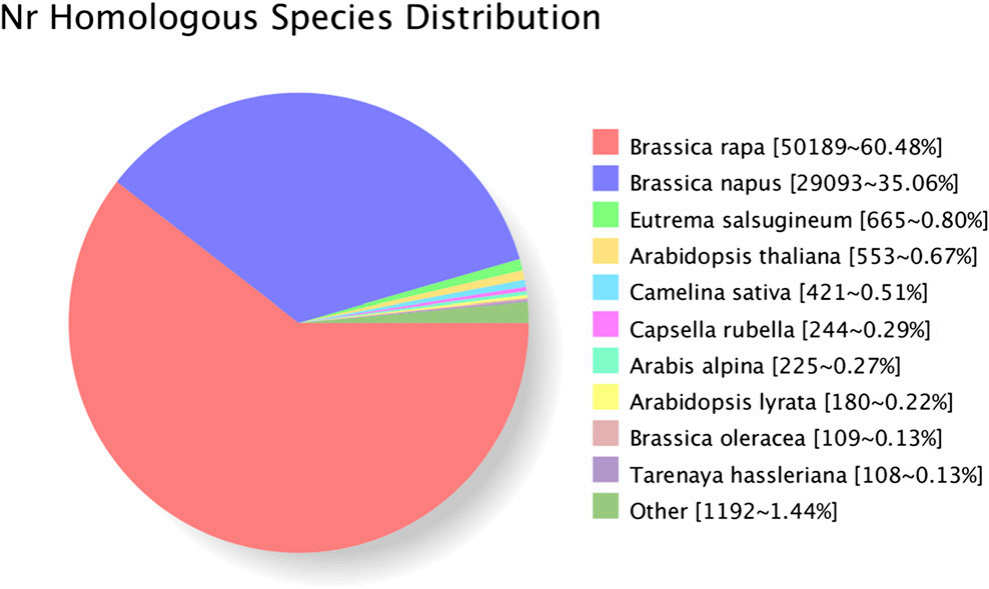
Homologous species distribution of *Brassica rapa var. Rapa* transcripts annotated in the NR database.

**Figure 6:**
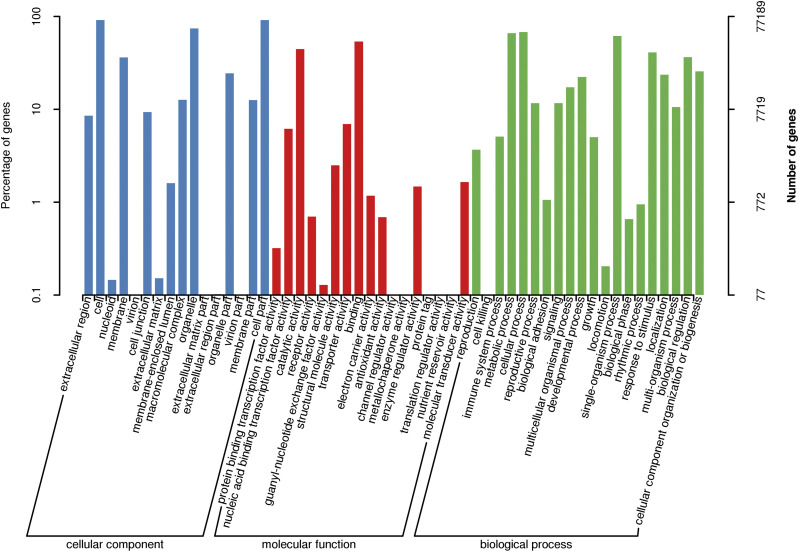
Gene ontology (GO) functional annotation of *Brassica rapa var. Rapa* transcripts Green represents biological process, blue represents molecular function, and red represents cellular component. The x-axis shows the GO categories, the y-axis (right) represents the number of transcripts, and the y-axis (left) represents the percentage of transcripts.

**Figure 7:**
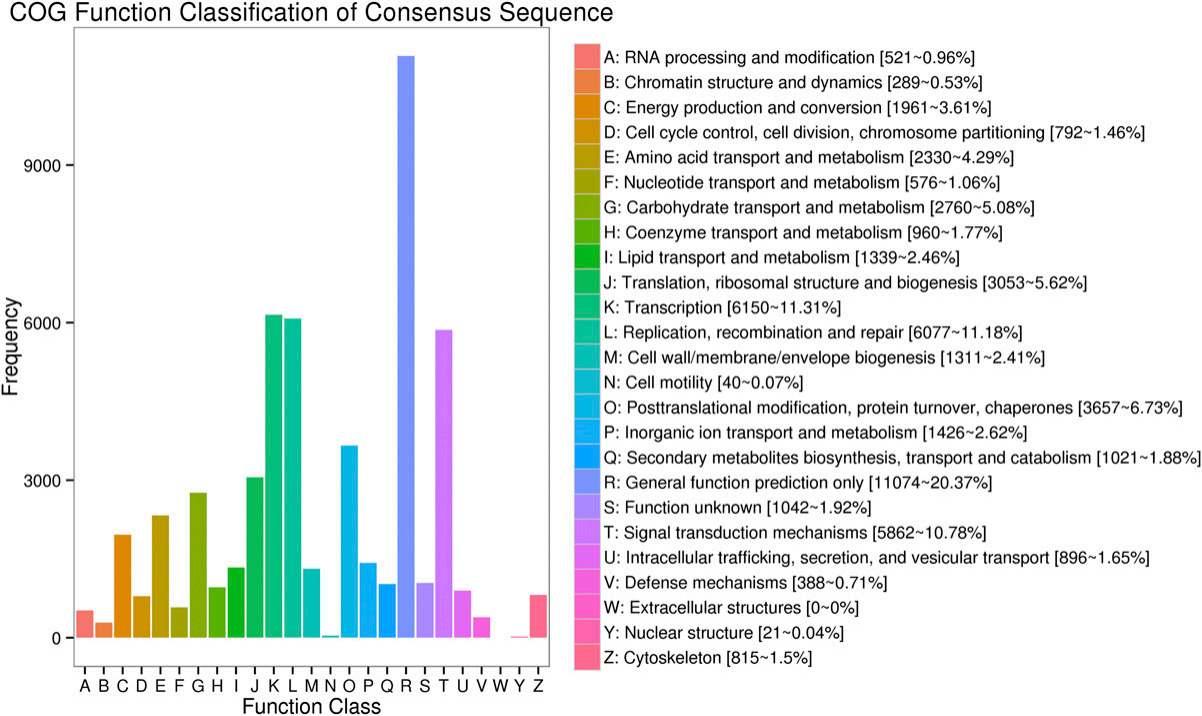
Clusters of orthologous groups of proteins (COG) annotation of *Brassica rapa var. Rapa* transcripts The x-axis shows the COG categories, and the y-axis represents the number of transcripts.

Functional annotations were performed for the 83,820 non-redundant transcripts. BLAST software (version 2.2.26) was used to compare the obtained transcript sequences with the NR, SwissProt, GO, COG, KOG, Pfam, and KEGG databases to obtain annotation information for the transcripts. In total, 35,506 transcripts were annotated in the COG database, 77,189 in the GO database, 35,918 in the KEGG database, 53,357 in the KOG database, 70,213 in the Pfam database, 65,292 in the SwissProt database, 80,379 in the eggNOG database, and 82,983 in the NR database. Moreover, 83,065 transcripts were annotated in at least one of the eight databases (Table 5). In NR annotation, the species with the sequence most homologous to each *Brassica rapa var. Rapa* transcript were predicted. The greatest number of sequences, approximately 60.48%, aligned to *Brassica rapa*, followed by *Brassica napus* (35.06%), *Eutrema salsugineum* (0.80%), *Arabidopsis thaliana* (0.67%), and *Camelina sativa* (0.51%) (Figure 5). In GO annotation, the transcripts were significantly enriched in 20 biological processes terms, 17 molecular function terms, and 16 cellular component terms (Figure 6). The results showed that most of the transcripts were mainly functioned in biological processes including cellular process, metabolic process, single-organism process, response to stimulus, biological regulation. Catalytic activity, binding and transporter activity were the major molecular functions of the transcripts. The cellular component functions of those transcripts were mainly cell part, cell and organelle. The COG database is based on bacteria, algae, and the system evolution of eukaryote relationship building. In COG annotation, the transcripts were most enriched in function R (general function prediction only, 20.37%), followed by function K (transcription, 11.31%) and function L (replication, recombination and repair, 11.18%) (Figure 7).

**Table 5:** Transcripts annotation statistics

Anno_Database	Annotated number	Length (300-1000)	Length (≥1000)
COG_Annotation	35506	3433	32073
GO_Annotation	77189	8147	69042
KEGG_Annotation	35918	4064	31854
KOG_Annotation	53357	5431	47926
Pfam_Annotation	70213	6820	63393
Swissprot_Annotation	62592	6126	56466
eggNOG_Annotation	80379	8604	71775
NR_Annotation	82983	9020	73963
All_Annotated	83065	9035	74030

### Prediction of lncRNAs

**Figure 8:**
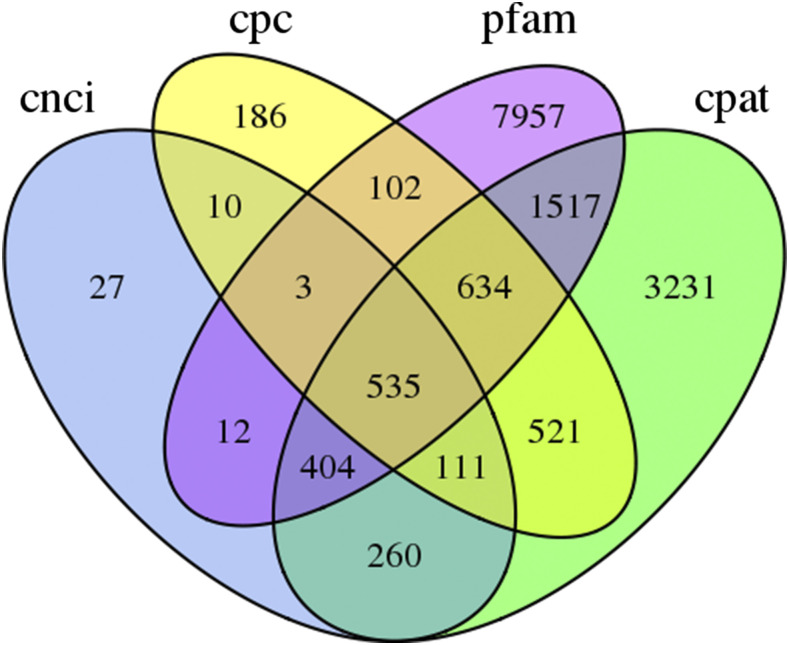
Venn diagram of the number of lncRNAs predicted by Coding Potential Calculator (CPC), Coding-Non-Coding Index (CNCI), Coding Potential Assessment Tool (CPAT), and Pfam Protein Structure Domain Analysis.

LncRNAs are not protein-coding, and as a result, screening transcripts for coding potential can identify potential lncRNAs. Based on the four methods described above, a total of 535 lncRNA transcripts were predicted (Figure 8). In addition, the target transcripts of the lncRNAs were further predicted using the LncTar tool, and 92 lncRNA were found to target at least one transcript (Table S3).

### Transcript mapping to field mustard (Brassica rapa)

In SMRT sequencing, a total of 46,516 transcripts and 49,429 transcripts were obtained for Xinjiang green turnips (F01) and Xinjiang purple turnips (F02), respectively. Based on the method described above, these transcripts were mapped to field mustard (*Brassica rapa*) to identify potential novel transcripts. For Xinjiang green turnips (F01), 41,322 known transcripts were detected, 810 transcripts of which completely mapped (100% mapping and matching rate) to field mustard (*Brassica rapa*), and 5,194 novel transcripts were obtained. Similarly, for Xinjiang purple turnips (F02), 44,060 known transcripts were detected, 1,199 transcripts of which completely mapped (100% mapping and matching rate) to field mustard (*Brassica rapa*), and 5,369 novel transcripts were obtained (Table 6).

**Table 6:** Statistics of transcripts mapping to field mustard (*Brassica rapa*)

mapping rate		matching rate				
		100%	90–100%	80–90%	70–80%	total
100%	F01	810	12335	814	381	14340
	F02	1199	13400	917	355	15871
90–100%	F01	924	21452	1354	492	24222
	F02	1364	21986	1451	517	25318
80–90%	F01	128	1538	224	80	1970
	F02	158	1574	227	80	2039
70–80%	F01	54	542	128	66	790
	F02	82	567	112	71	832

## discussion

In recent years, transcriptome research has greatly accelerated due to the high accuracy of short reads generated by next generation sequencing. Nevertheless, short reads shows lower contiguity in sequence assembly, making analysis of complex genomic regions and specific biological processes difficult (Hackl *et al.* 2014; Koren *et al.* 2012). SMRT sequencing, which exhibits more advantages in transcriptome research than short-read sequencing due to the generation of full-length transcripts, has been comprehensively investigated (Sharon *et al.* 2013). Despite the relatively high error rate of third-generation sequencing, this disadvantage can be corrected by accurate short reads (Dong *et al.* 2015). In our current study, 267,666 FLNC reads were obtained from Xinjiang green turnip (F01). In addition, 82,640 consensus transcript sequences were obtained in sequence clustering analysis, of which 69,480 were high-quality, and the 13,160 low-quality sequences were corrected using Illumina RNA-seq data. Similarly, a total of 274,915 FLNC sequences and 93,775 consensus transcript sequences (14,977 corrected sequences) were obtained from Xinjiang purple turnip (F02).

Following the removal of redundant sequences, there were 46,516 and 49,429 non-redundant transcripts for F01 and F02, respectively. A series of annotation analyses were performed on those transcripts. In NR annotation, the species with the sequence most homologous to each *Brassica rapa var. Rapa* transcript was predicted. 60.48% of sequences aligned to *Brassica rapa*, followed by *Brassica napus* (35.06%). Both *Brassica rapa* and *Brassica napus* belong to the Brassica species. *Brassica rapa* (AA, 2n = 20), *Brassica oleracea* (CC, 2n = 18), and their allopolyploid derivative, *Brassica napus* (AACC, 2n = 38) are the three most well known Brassica crops (Qi *et al.* 2017; Wang *et al.* 2011). Each of these Brassica species includes several cultivated subspecies that were domesticated for different use with diverse morphological characteristics. It has been considered in a long time as classic textbook example of artificial selection during plant domestication and breeding (Wang *et al.* 2011; Cheng *et al.* 2016; Qi *et al.* 2017). Wang *et al.* annotated and analyzed of the draft genome sequence of *Brassica rapa ssp. pekinesis* Chiifu, and modeled 41,174 protein coding genes in the *B. rapa* genome (Wang *et al.* 2011). They suggested that the remarkable morphological plasticity of Brassica species was prpbably benefitted from the variation in the number of gene families members occurred in genome. Notably, the obtained transcripts for F01 and F02 were mapped to the *Brassica rapa* genome, and 5,194 and 5,369 novel transcripts were identified. These might be explained the morphological difference of *Brassica rapa ssp. Rapa* from other *Brassica rapa* crops.

AS is one of the common ways to diversify the functional characteristics of the transcriptome and the proteome in eukaryotic organisms (Reddy *et al.* 2013; Kalsotra and Cooper 2011). AS is implicated in the regulation of plant development, as it occurs in approximately 40–60% of intron-containing transcripts in different tissues and developmental stages in *Arabidopsis thaliana* (Marquez *et al.* 2012), *Zea mays* (Thatcher *et al.* 2014), and Oryza sativa (Dong *et al.* 2018). In Xinjiang green turnip (F01) and purple turnip (F02), a total of 620 and 872 AS events were predicted, respectively. Non-coding RNAs (ncRNA) refer to RNAs that lack the ability to encode proteins, and were initially regarded as inessential transcriptional “noise.” Research advances have demonstrated the crucial regulatory roles of ncRNAs in various biological processes (Do *et al.* 2019). Plant lncRNAs had been reported to participate in photomorphogenesis, auxin transport, flowering, etc. (Yang *et al.* 2020; Shafiq *et al.* 2016; Liu *et al.* 2015). Based on the four commonly used methods described above, a total of 535 lncRNA transcripts were predicted in our study. In addition, the target transcripts of 92 lncRNAs were further predicted. Nevertheless, further study is needed to understand the functions and biological processes they are involved in.

## Conclusion

In conclusion, the full-length transcriptome of *Brassica rapa var. Rapa* was first investigated using SMRT sequencing, which may facilitate further studies into the genetic data of *Brassica rapa var. Rapa* and may help to clarify the annotation of the turnip genome as well as serve as a reference for other brassica species. The results of this study are of great significance to further study the dynamic changes in transcription and the differential expression of transcripts during the growth and development of turnips.
